# Multiplex amplicon sequencing for the comprehensive genotyping of *Mycoplasma pneumoniae*

**DOI:** 10.1128/spectrum.02719-24

**Published:** 2025-05-22

**Authors:** Hiroaki Kubota, Rumi Okuno, Tsuyoshi Kenri, Yumi Uchitani, Tsukasa Ariyoshi, Isao Yoshida, Kai Kobayashi, Morika Mitobe, Jun Suzuki, Kenji Sadamasu

**Affiliations:** 1 Department of Microbiology, Tokyo Metropolitan Institute of Public Health, Tokyo, Japan; 2 Department of Bacteriology II, National Institute of Infectious Diseases, Tokyo, Japan; ARUP Laboratories, Salt Lake City, Utah, USA

**Keywords:** *Mycoplasma pneumoniae*, genotypic identification, molecular epidemiology, phylogenetic analysis, genome analysis, antibiotic resistance

## Abstract

Major genotyping methods used to characterize *Mycoplasma pneumoniae* strains are based on various experimental approaches that need to be implemented in parallel for each strain. In this study, we developed a comprehensive workflow based on amplicon sequencing using next-generation sequencing. This workflow comprised PCR amplification with seven tubes, collection into single tubes, shotgun sequencing, *de novo* assembly separating each target into individual contigs, and *in silico* genotyping. The results for *p1*, *orf6*, multilocus sequence types, 23S ribosomal RNA gene mutations conferring macrolide resistance, and single-nucleotide polymorphisms identifying the *p1* type 1 lineage were obtained simultaneously. The genotyping accuracy was confirmed by comparing the sequences with the whole-genome sequences of 40 *M*. *pneumoniae* isolates collected in Tokyo, Japan. The workflow described not only enables high-throughput comprehensive data collection but also enables the detection of novel genotypes with single-nucleotide resolution.

**IMPORTANCE:**

Genotyping plays a central role in the molecular epidemiology of pathogenic bacteria, and many methods have been developed to identify prevalent lineages, infection routes, and antimicrobial resistance. Whole-genome sequencing generally provides most of the genetic information targeted by classic PCR-based schemes and has contributed to the construction of a simplified workflow for many bacterial species. However, several issues concerning the *Mycoplasma pneumoniae* genome, such as the presence of repetitive elements of the *p1* gene, prevent the collection of genotyping results from a single run of short-read shotgun sequencing. Herein, we describe a simplified workflow using amplicon sequencing that covers most of the major genotyping schemes for *M. pneumoniae*, including *p1* genotyping. The workflow effectively characterized *M. pneumoniae* clinical isolates. This workflow could help advance research on the molecular epidemiology of *M. pneumoniae* and the detection of novel genotypes.

## INTRODUCTION

The epidemiology of *Mycoplasma pneumoniae* is characterized by periodic epidemics that occur at intervals of several years. Although periodicity was likely disturbed during the COVID-19 pandemic, efforts to monitor *M. pneumoniae* have continued worldwide (1). The prevalent *M. pneumoniae* lineages observed by genotyping tend to be associated with periodic epidemics; for example, *p1* type 1 lineage was disseminated around 2012 in the eastern regions of Japan, and the predominance of *p1* type 2c was observed around 2016 (2, 3). This phenomenon has also been associated with a sequence type (ST) change, in multilocus sequence typing (MLST) analysis, and a decrease in the prevalence of macrolide-resistant strains (4).

The characterization of the prevalent *M. pneumoniae* lineage is important, and several genotyping methods have been developed and used for decades (5). The *p1* operon, which contains the *p1* and *orf6* genes for P1 and P40/P90 proteins, is a major target of *M. pneumoniae* genotyping (6). P1 and P40/P90 are components of the adhesin complex (nap) and play pivotal roles in infection (7). The *p1* and *orf6* genes exhibit sequence variations in their repetitive element regions (RepMP4, RepMP2/3, and RepMP5) (7, 8). Although the RepMP4 and RepMP2/3 regions in the *p1* gene have been widely used for genotyping because of their diversity, named the *p1* genotype, the RepMP5 region in the *orf6* gene, which encodes P40/P90, has also been reported to have unique variations characterizing *M. pneumoniae* lineages. Specifically, *p1* type 2a strains were separated into two lineages that shared *orf6* sequences with types 2 and 2c strains (2, 9).

The highest resolution of genotyping can be achieved through whole-genome analysis using next-generation sequencing (NGS). As demonstrated in various bacterial species, a comparison of single-nucleotide polymorphisms (SNPs) can distinguish the lineages by clarifying their phylogenetic relationships. Furthermore, NGS analysis can simultaneously provide most of the typing results based on the classical genotyping scheme of strains, reducing the time and effort of individual analyses. For example, MLST, generally implemented by PCR amplification and Sanger sequencing of typically five to eight target regions, can be simultaneously performed in the same run used for SNP comparisons (10). In addition, other clinically important genes, such as antimicrobial resistance or virulence-associated genes, can be detected at the same time (11, 12). In some cases, several representative SNPs reflecting the lineages, recognized in the whole-genome analysis, have been extracted and applied to classical PCR-based methods for pseudo-whole-genome analysis (13, 14).

However, whole-genome analysis is not necessarily superior to classic PCR-based methods. When whole-genome data were acquired using a short-read sequencer such as MiSeq (Illumina, San Diego, CA, USA), repetitive elements, such as RepMP4, RepMP2/3, and RepMP5 regions of *p1* and *orf6*, were hardly assembled to reconstruct the original sequences. In molecular epidemiological studies of *M. pneumoniae*, *p1* and *orf6* gene sequences are important for strain characterization. Therefore, the simple application of whole-genome short-read shotgun sequencing using NGS is not ideal (15). The *p1* and *orf6* genotypes frequently cannot be identified from draft genome sequences in public databases. The hybrid assembly of short reads with long reads is capable of reconstructing such repetitive elements; however, the long-read data must be acquired with another run using long-read sequencers, such as MinION (Oxford Nanopore, Oxford, UK).

**Fig 1:**
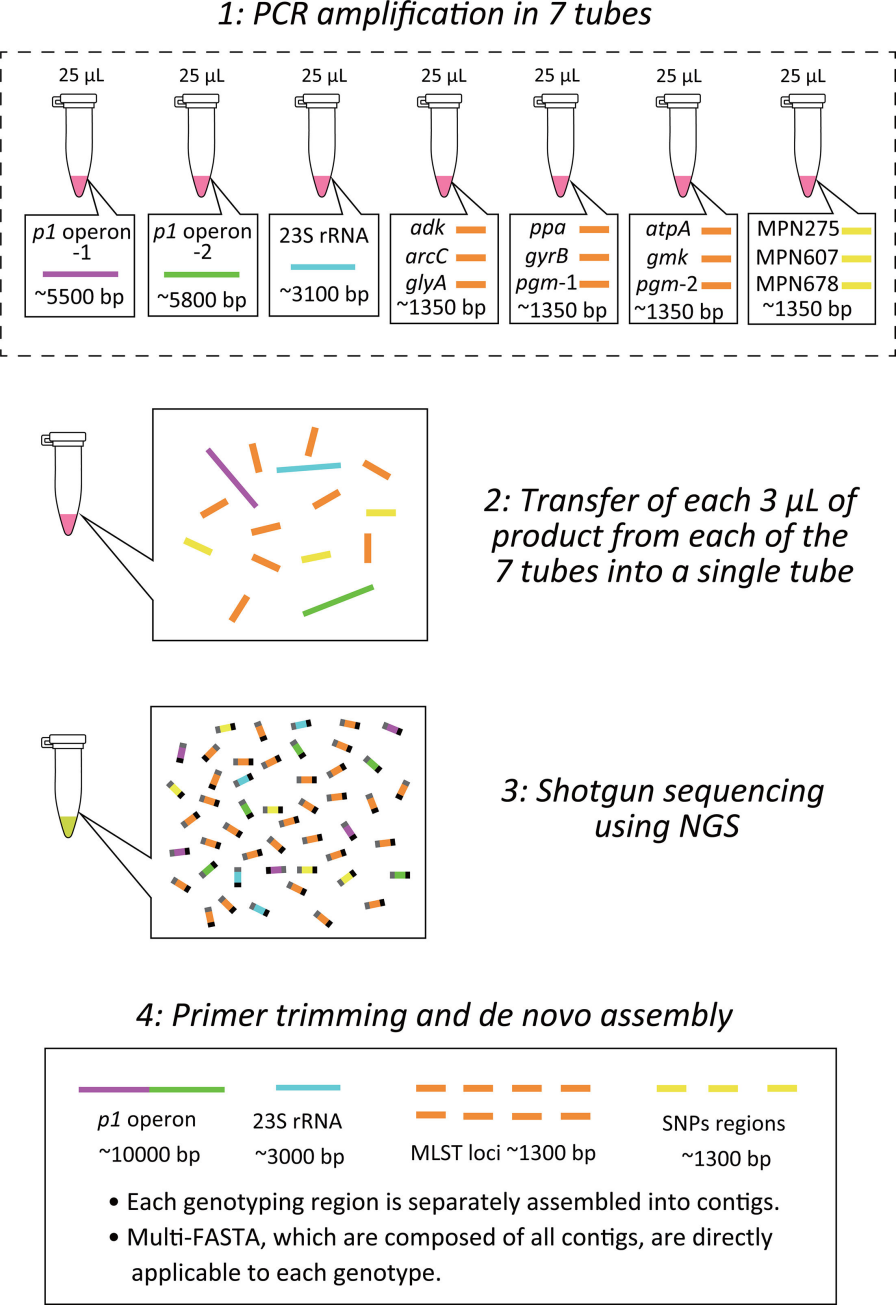
Schematic representation of the genotyping workflow.

In this study, we developed a simplified but comprehensive workflow covering most of the major genotyping methods for the molecular epidemiology of *M. pneumoniae* (Fig. 1), including *p1* and *orf6* genotyping (8), MLST (16), detection of 23S rRNA gene mutations (17), and identification of representative SNPs in *p1* type 1 lineage (8). Amplicon sequencing was performed to facilitate the sequencing of *p1* and *orf6*. Additionally, the total number of target sequences (approximately 30,000 bp) was approximately 25-fold shorter than the whole genome of *M. pneumoniae* (approximately 800,000 bp). Therefore, many samples can be analyzed in one NGS run, or sequencing kits output fewer data, which incurs a lower cost and shorter running time. Moreover, as the analyses are based on sequencing, our workflow enables the emergence of new variants of *p1* and *orf6* genes and new MLST types to be detected. The accuracy of the data output of this workflow was evaluated by comparing it with that obtained by whole-genome analysis, including complete genome sequences reconstructed by the hybrid assembly.

## MATERIALS AND METHODS

### Strains

Forty *M. pneumoniae* strains were isolated from nasopharyngeal swab specimens collected from five hospitals in Tokyo between 2016 and 2023. First, according to the national epidemiological surveillance protocol for infectious diseases, nasopharyngeal swabs collected from patients with suspected *M. pneumoniae* infection were dipped into 2 mL growth medium (21 g/L Difco PPLO broth [Thermo Fisher Scientific, Waltham, MA, USA], 0.002% phenol red, 15% heat-inactivated fetal horse serum, 2.5% yeast extract, and 1% glucose) supplemented with 1,000 units/mL penicillin G and 1,000 units/mL vancomycin at the hospitals, and stored at −20°C until the transfer to our laboratory. After transfer, the parts of samples were inoculated onto fresh growth medium and incubated for weeks at 37°C. The bacterial growth was inoculated onto the Difco PPLO agar (Thermo Fisher Scientific) plate containing 15% heat-inactivated fetal horse serum, 2.5% yeast extract, and 1% glucose to isolate single colonies. The incubation in growth medium was repeated, and the growth of *M. pneumoniae* was confirmed using a Loop-Mediated Amplification Kit (Eiken Kagaku, Tokyo, Japan). The details of the isolates are summarized in Table 1.

**TABLE 1:** Strains used in this study and summary of the assembly of amplicon sequencing^*d*^

Strain	Sample collection year	Patient age (years)	Hospital	*p1* operon				23S rRNA mutation	MLST	*p1* type 1 lineage SNPs			
				RepMP4	RepMP2/3	*p1* type^*a*^	orf6			MPN295 G135T	MPN607 A465G	MPN678 C435T	*p1* type 1 clade^*b*^
*p1* type 1													
TA6243	2016	6	A	1	1/1b	**1**	1**^*c*^**	A2063G	3	+	+	−	**T1-3R**
TA6350	2016	6	B	1	1/1b**^*c*^**	**1**	1**^*c*^**	A2063G	3	−	+	−	**T1-3**
TA6349	2016	3	B	1	1/1b	**1**	1**^*c*^**	A2063G	3	+	+	−	**T1-3R**
TA6366	2016	9	A	1	1/1b	**1**	1**^*c*^**	A2063G	3	+	+	−	**T1-3R**
TA6381	2016	3	B	1	1/1b	**1**	1**^*c*^**	A2063G	3	+	+	−	**T1-3R**
TA6411	2016	7	C	1	1/1b	**1**	1**^*c*^**	A2063G	3	+	+	−	**T1-3R**
TA6425	2016	6	B	1	1/1b	**1**	1**^*c*^**	A2063G	3	+	+	−	**T1-3R**
TA6472	2016	9	A	1	1/1b**^*c*^**	**1**	1**^*c*^**	A2063G	3	+	+	−	**T1-3R**
TA6464	2016	5	A	1	1/1b	**1**	1**^*c*^**	A2063G	30	−	+	−	**T1-3**
TA6512	2016	17	B	1	1/1b	**1**	1**^*c*^**	A2063G	3	+	+	−	**T1-3R**
TA6580	2016	14	A	1	1/1b	**1**	1**^*c*^**	A2063G	3	+	+	−	**T1-3R**
TA9076	2019	17	E	1	1/1b	**1**	1**^*c*^**	A2063G	3	+	+	−	**T1-3R**
TA9171	2019	5	E	1	1/1b**^*c*^**	**1**	1**^*c*^**	wt	17	−	+	+	**T1-2**
TA9276	2019	9	E	1	1/1b	**1**	1**^*c*^**	A2063G	3	+	+	−	**T1-3R**
TA9308	2019	14	E	1**^*c*^**	1/1b	**1**	1**^*c*^**	A2063G	3	+	+	−	**T1-3R**
TA9312	2019	3	E	1	1/1b	**1**	1**^*c*^**	A2063G	3	+	+	−	**T1-3R**
TA9349	2019	4	E	1	1/1b	**1**	1**^*c*^**	A2063G	3	+	+	−	**T1-3R**
TA9468	2020	10	E	1	1/1b	**1**	1**^*c*^**	A2063G	3	+	+	−	**T1-3R**
NA2046	2023	9	E	1	1/1b	**1**	1**^*c*^**	A2063G	3	+	+	−	**T1-3R**
*p1* type 2													
TA6463	2016	37	B	2c/2f	2c	**2c**	2c	wt	14	−	+	−	N/A
TA6494	2016	11	D	2c/2f	2c	**2c**	2c	wt	14	−	+	−	N/A
TA6778	2017	13	A	2c/2f	2c	**2c**	2c	wt	14	−	+	−	N/A
TA7396	2017	18	E	2c/2f**^*c*^**	2c	**2c**	2c	wt	14	−	+	−	N/A
TA7750	2018	6	E	2c/2f	2c**^*c*^**	**2c**	2c	wt	14	−	+	−	N/A
TA8574	2018	7	E	2c/2f	2c	**2c**	2c	wt	14	−	+	−	N/A
TA8674	2018	9	E	2c/2f	2c	**2c**	2c	wt	14	−	+	−	N/A
TA8743	2019	2	E	2j	2j	**2j**	2	wt	7	−	+	−	N/A
TA9139	2019	6	E	2j	2j	**2j**	2	wt	7	−	+	−	N/A
TA9221	2019	10	E	2j	2j	**2j**	2	wt	7	−	+	−	N/A
TA9241	2019	10	E	2c/2f	2c	**2c**	2c	wt	14	−	+	−	N/A
TA9242	2019	10	E	2j	2j	**2j**	2	wt	7	−	+	−	N/A
TA9307	2019	8	E	2c/2f	2c	**2c**	2c	wt	14	−	+	−	N/A
TA9309	2019	0	E	2c/2f	2c	**2c**	2c**^*c*^**	wt	14	−	+	−	N/A
TA9313	2019	4	E	2c/2f	2c	**2c**	2c	wt	14	−	+	−	N/A
TA9336	2019	12	E	2c/2f	2c	**2c**	2c	wt	14	−	+	−	N/A
TA9337	2019	8	E	2j	2j	**2j**	2	wt	7	−	+	−	N/A
TA9338	2019	8	E	2j	2j	**2j**	2	wt	7	−	+	−	N/A
TA9509	2020	8	E	2j	2j	**2j**	2	wt	7	−	+	−	N/A
TA9565	2020	11	E	2j	2j	**2j**	2	wt	7	−	+	−	N/A
TA9617	2020	14	E	2c/2f**^*c*^**	2c	**2c**	2c**^*c*^**	wt	14	−	+	−	N/A

^*a*^ The *p1* type was determined by the combination of RepMP4 and RepMP2/3 types, using the PCR-RFLP scheme (6).

^*b*^ The clades were determined by the combination of three SNPs for *p1* type 1 strains.

^*c*^ Sequences not identical to the listed references.

^*d*^ N/A, not applicable; PCR, polymerase chain reaction; RFLP, restriction fragment length polymorphism; SNP, single-nucleotide polymorphism; wt, wild type; +, presence; -, absence.

### Amplicon sequencing

The *M. pneumoniae* strains incubated in 2–10 mL of growth medium were collected by centrifugation at 20,000 × *g* for 5 min and the DNA was extracted using the QIAamp DNA Mini Kit (QIAGEN, Hilden, Germany). PCR amplification was performed in two tubes for the *p1* operon, one tube for the 23S rRNA gene, three tubes for the eight loci of MLST (*ppa*, *pgm*, *gyrB*, *gmk*, *glyA*, *atpA*, *arc*, and *adk*), and one tube for the three regions (MP295, MP607, and MP678) for SNP typing. The sequences and the concentrations for each mixture are shown in Table 2. Each tube was used for 25 µL-volume reactions: 12.5 µL PrimerSTAR GXL Premix (Takara Bio, Shiga, Japan), 1 µL primer mix, 0.25 µL template DNA, and 11.25 µL distilled water. The PCR conditions for *p1* operon and 23S rRNA gene were 30 cycles at 98°C for 10 s, 60°C for 15 s, and 68°C for 6 min, and those for MLST and SNPs typing were 35 cycles at 98°C for 10 s, 60°C for 15 s, and 68°C for 90 s. After amplification, 3 µL of the product was collected from each tube and united in a single tube per strain, followed by purification using AMpure XP Magnetic Beads (Beckman Coulter, Brea, CA, USA) according to the manufacturer’s protocol. The united and purified amplicons per strain were used for library preparation using the Nextera XT Library Preparation Kit (Illumina) according to the manufacturer’s protocol. The sequencing was performed on the MiSeq platform (Illumina) using the MiSeq Reagent Nano Kit 500 Cycles (Illumina) to obtain 2 × 250 bp paired-end reads. The acquired read data and Nextera adapter sequences were trimmed to quality (>20) using Trim_Galore v0.6.7 (https://github.com/FelixKrueger/TrimGalore), followed by primer trimming (Table 2) on cutPrimers (18) for the *p1* operon and *pgm* regions with default settings. Subsequently, *de novo* assembly was performed using Spades v3.15.5 (19) with the “meta” option setting enabled and the other settings on default (20) to collect contig sequences as a multi-FASTA file.

**TABLE 2:** PCR primers used in this study^*b*^

Mixture (concentration)	Primer name	Sequence (5′ to 3′)	Product length^*a*^
*p1* operon 1 (10 µM)	p1_comp_1F	CTTCATAAAAACTGCCAACCAAGTT	5,487 bp
	p1_comp_1R	TCATACACCAACATAGTTACCGGATC	
*p1* operon 2 (10 µM)	p1_comp_2F	CTGTCATTCACCAACAAGAACAAC	5,784 bp
	p1_comp_2R	GCTTGAAGCCCATTAAAATCTTTATG	
23S rRNA (10 µM)	23S_comp_F	CTGAAAACGACAATCTTTCTAGTTCC	3,139 bp
	23S_comp_R	AGACCTAGGCATAGGCCTAAGTCTC	
MLST 1 (5 µM)	adk_comp_F	TTGTCCTGTATGGTATCTTGGTGATT	1,349 bp
	adk_comp_R	CTTAACACCAAAAACACGGTTGTC	
	arcC_comp_F	ATGGAAGTGACTGATACGGTGTTT	1,355 bp
	arcC_comp_R	AAGGTCAACGGTAGGTACAAATAGG	
	glyA_comp_F	AACGTTCTAACAGTTTTAAAACCGC	1,351 bp
	glyA_comp_R	GCAGCATTGGTTATGATCTTATCTTT	
MLST 2 (5 µM)	ppa_comp_F	CCAAATAAAGCTACAAAGTGACAGC	1,318 bp
	ppa_comp_R	TTGTGCTTTTTGATCAATAAGGTCAC	
	gyrB_comp_F	CTAACCCTTAAAGACAACTTTGTCACC	1,350 bp
	gyrB_comp_R	CATCGGTCATGATAATGATCTTGTT	
	pgm_comp_1F	TTGTTAACGGTAATTTCCTTAATTTCCT	1,404 bp
	pgm_comp_1R	TAAGTTGCACTTAATTGGTCTGTTTTC	
MLST 3 (5 µM)	atpA_comp_F	TTACCGTTGGGTTGTTTAATCATC	1,325 bp
	atpA_comp_R	TCCGTGATCAACTATCACGTTTAAT	
	gmk_comp_F	GATCTCTTTTTGTTGGAGTCAATCA	1,354 bp
	gmk_comp_R	GCAAACTGGACTTGGTTACGTTTAAT	
	pgm_comp_2F	GGCCACAATAATGACATCACTCTTAAT	1,440 bp
	pgm_comp_2R	TGGGACAGAGAAGAGATTGCTTATAA	
SNP typing (5 µM)	295_comp_F	CAACACTTTGTCCAAAACTAACTTTAACC	1,398 bp
	295_comp_R	TGGTAAAATTAAAGCGATGGTTAGTTAAC	
	607_comp_F	TCCAGTATGACCACTAGCTAACTTCAC	1,370 bp
	607_comp_R	CAAAGCAAAAGGAAACTAACCGTTA	
	678_comp_F	GGTTCTTAAAACAGTGTTTTCACCAG	1,329 bp
	678_comp_R	CAAAGGTTTCCTTAATCAGTTGGTTAATT	

^*a*^ The product lengths shown are those of the amplicon in M129 (U00089.2).

^*b*^ MLST, multilocus sequence typing; SNP, single-nucleotide polymorphism.

### *In silico* genotyping

For the genotyping of RepMP4, RepMP2/3, and *orf6*, reference sequences for each region (Table S1) were searched on the multi-FASTA files using a MegaBlast algorithm, and point mutations in the 23S rRNA gene, MPN295, MPN607, and MPN678 were detected in a similar manner. When the contigs for each region in the *p1* operon (RepMP4, RepMP2/3, and *orf6*) were not 100% identical to the listed references, the closest matching genotypes were determined by sequence alignment and construction of a neighbor-joining tree using MEGA7 software. The ST was determined by running the MLST program (https://github.com/tseemann/mlst) on the multi-FASTA file.

### Whole-genome sequencing

The extracted DNA was used for library preparation using the Nextera XT Library Preparation Kit (Illumina) according to the manufacturer’s protocol and sequenced on the MiSeq platform (Illumina) using the MiSeq Reagent Kit v3 600 Cycles (Illumina) to obtain 2 × 300 bp paired-end reads. The acquired read data in FASTQ format were applied to quality (>20) and adapter trimming by Trim Galore and *de novo* assembled using Spades with the default settings to obtain contigs for comparison with the data obtained from the amplicon sequencing. These short reads were used for subsequent phylogenetic analyses or hybrid assembly with long-read data.

For long-read sequencing, a library was prepared using the Native Barcoding Kit 24 V14 (Oxford Nanopore) according to the manufacturer’s protocol and sequenced on the MinION platform using an R10.4.1 flow cell (Oxford Nanopore). The data were extracted using the MinKNOW program with a SUP model. The hybrid assembly of these long reads with short reads obtained as described above was implemented using Hybracter v0.7.3 (21) with default settings. The complete genome sequence of each strain was annotated using the DFAST website (https://dfast.ddbj.nig.ac.jp/) and deposited in the DNA Data Bank of Japan.

### Phylogenetic analysis

The trimmed paired-end short reads of the strains collected in Tokyo were mapped onto strain M129 (U00089) or 309 (AP012303), in case of *p1* types 1 and 2 lineages, respectively, using Snippy v4.6.0 (https://github.com/tseemann/snippy) with default settings. Similarly, the complete and draft genome sequences available in the public database for July 2024 (Table S2), from which *p1* and *orf6* genotypes were successfully determined from the surrounding sequences, were also aligned to these reference genomes. A whole-genome alignment file was created from these Snippy output data, using Snippy-core v4.6.0 (https://github.com/tseemann/snippy), followed by SNP calling and construction of a neighbor-joining tree with the putative recombination sites removed using Gubbins v3.3.1 (22) with default settings. Tree visualization was performed using the TVBot website (23).

## RESULTS

### Assembly of contigs of 40 strains by comprehensive workflow

Our comprehensive workflow outputs all contigs of 40 strains (Table S3). The concentration of input DNA to PCR was broadly ranged (0.094–106 µg/mL), without lacking any data collection from all strains. This demonstrated that the input DNA concentration did not significantly affect the results. Among the eight MLST loci, the *pgm* locus was amplified using two PCR primers, and the two amplicons were merged during *de novo* assembly. We used two PCR reactions because our initial attempt with a single PCR (not shown here) frequently provided insufficient length of assembled contigs on *pgm* typing, probably due to the longer target region (~1,200 bp) than the other seven loci (<1,000 bp). Examples of contig sequences for the two strains, TA6243 and TA6463, are provided in Table S4.

### Genotyping of the 40 strains

Each genotype (ST, 23S rRNA gene, MPN295, MPN607, and MPN678) determined from the comprehensive workflow was identical to that obtained from the assembled contigs of the paired-end short reads. We tested this process with standalone and web-based Basic Local Alignment Search Tool search (https://blast.ncbi.nlm.nih.gov/Blast.cgi) using the “align two or more sequences” mode and confirmed that both the methods worked correctly. Likewise, the standalone MLST was consistent with the analysis on the pubMLST website (https://pubmlst.org/). Sequences similar but not identical to the listed reference sequences (Table S1) were found in the RepMP4 region of three strains, the RepMP2/3 region of four strains, and the *orf6* region of 21 strains (Table 1). If these sequence variations were generated by recombination between the *p1* operon locus and external RepMP elements, it would be necessary to assign them as new genotypes (8). However, inspections of RepMP sequences of complete genome sequences obtained in this study showed that all the new sequences could be classified according to known genotypes. The variations identified in this study may have arisen spontaneously in the *p1* operon without RepMP recombination (Fig. S1). Therefore, strains that included such variations, were classified as general *p1* or *orf6* types. Among them, new sequences for several strains that did not match any *M. pneumoniae* sequences in the GenBank database, owing to several nucleotide variations, were found in RepMP4 from strains TA7396, TA9308, and TA9617; RepMP2/3 from strains TA6350, TA6464, TA7750, and TA9171; and *orf6* from strains TA6464, TA9309, and TA9617. We confirmed that these nucleotide variations did not originate from an error in our workflow by comparing them with the complete genome sequences obtained by the hybrid assembly.

### Relationship between phylogenetic positions and genotyping profiles

**Fig 2:**
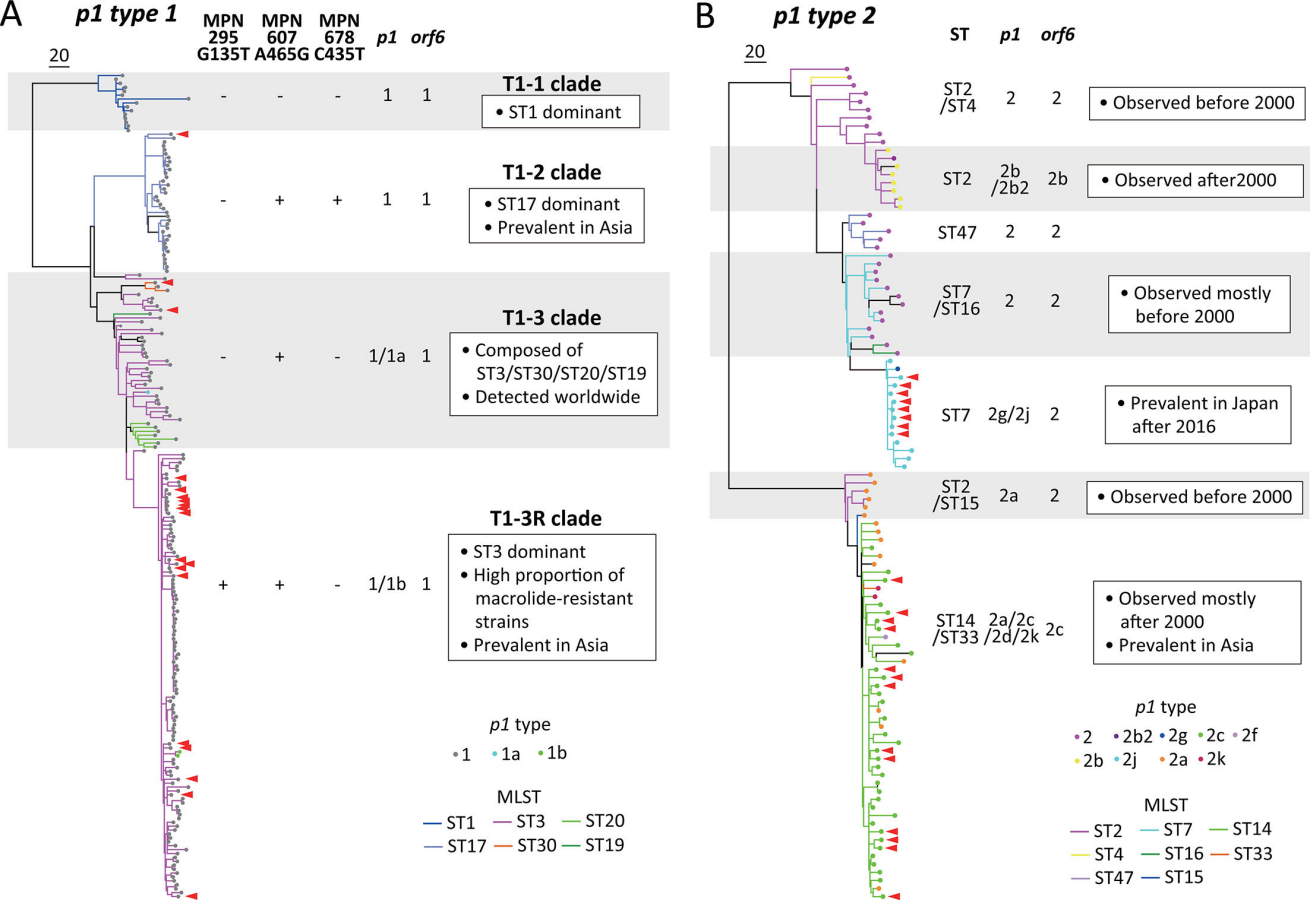
Phylogenetic tree showing the phylogenetic relationship between the different clades. The SNPs-based non-rooted tree for *p1* types 1 (**A**) and 2 (**B**) are shown with the clades specified by combination of genotypes which can be acquired by the comprehensive workflow. The *p1* and MLST genotypes were represented using colored plots and branches, respectively. Interpretations for each clade are summarized in boxes, and the clade names for type 1 tree are shown. Red arrowheads represent the positions of *M. pneumoniae* strains from Tokyo in this study. Scale bars = number of nucleotide substitutions. Full details of these trees are shown in Fig. S2 and S3.

The phylogenetic trees for *p1* types 1 and 2 lineages constructed (Fig. 2A and B) were consistent with previous reports (2, 8, 15, 24), and the strains added in this study did not create any novel branches. In these studies, *p1* type 1 could be sub-classified into several clades based on major branches that were indistinguishable based on their *p1* and *orf6* genotypes; however, the nomenclature for these clades has not been harmonized among the studies. In this study, we used the T1-1, T1-2, T1-3, and T1-3R classification methods (8) because the clustering of macrolide-resistant strains to the T1-3R clade is epidemiologically important (Fig. 2A). Most branches of the *p1* type 2 lineage reflected a combination of genotyping patterns of *p1*, *orf6*, and MLST (Fig. 2B). This method of classifying genetic relatedness among *M. pneumoniae* strains based on genotype combination is also effective for evaluating data from other researchers. For example, the recent metagenomic analysis of epidemic strains from China found that the EC2 strains were macrolide-resistant (25). Although the *p1* subtype of the EC2 strains was not mentioned in the report (25), our knowledge of genotype combination strongly suggests that the EC2 strains are part of *p1* type 2c and MLST ST14 strains, which is a prevalent genetic group of *M. pneumoniae* strains that have spread worldwide in recent years. Therefore, the data determined by a comprehensive genotyping workflow can be useful to interpret phylogenetic relationships of *M. pneumoniae* strains more precisely than the data analyzed using single genotyping methods.

## DISCUSSION

In this study, we developed a useful and powerful genotyping workflow to classify *M. pneumoniae* clinical isolates, enabling precise molecular epidemiological study of this species. The results obtained using this workflow were compatible with existing genotyping methods (6, 8, 16, 17) and provided an accurate classification of clinical isolates based on the phylogenetic relationships of this species (8). For classification of *M. pneumoniae* strains, the *p1* gene typing has mostly been used. *M. pneumoniae* strains were initially classified into two types (*p1* gene type 1 or 2 strains); however, the number of *p1* subtypes was increased step-by-step through detailed analyses of several clinical isolates by many researchers. At least 17 *p1* gene subtypes have been reported to date (8). The typing of *p1* is usually performed using standard PCR-restriction fragment length polymorphism (RFLP) analysis of the RepMP4 and RepMP2/3 regions of the *p1* gene (26). However, the resolution of RFLP analysis is limited and depends on the selection of restriction enzymes. Recently, some type 2 strains were reclassified as novel type strains with the type 2 j *p1* gene, because the 2 j *p1* gene could not be distinguished from classical type 2 *p1* by PCR-RFLP analysis using only *HaeIII* digestion (8). One cause of this problem was the difficulty in sequencing the *p1* operon in many strains. The *p1* operon is long (approximately 9 kb) and requires repeated Sanger sequencing to cover the entire region. In addition, the presence of repetitive elements (RepMP regions) makes *de novo* assembly of whole-genome sequencing data using NGS difficult (15). Our amplicon sequencing enabled sequence-level inspection of the *p1* operon using a relatively simple protocol and could prevent the oversight of new sequence variations that may sometimes occur in RFLP analysis.

During the evaluation of our genotyping workflow using the *M. pneumoniae* strains collected in Tokyo after 2016, we detected a difference in the prevalent STs from a previous report by Morozumi et al. 4, who analyzed *M. pneumoniae* strains collected at hospitals in Japan during the period 2002–2019. They reported that ST33 was dominant among *p1* type 2c strains during 2018–2019, shifting from ST14 which dominated during 2002–2016. However, ST33 was not detected in our study, and ST14 remained dominant even after 2016. One possible reason for this discrepancy is the differences in the study sites between the two studies. Morozumi et al. 4 reported that clinical samples were collected from 21 medical institutions throughout Japan and that the STs differed by area and year, but did not specify the exact locations of the study sites. The ST33 and ST14 strains differed in a single SNP located at the primer position of the *adk* marker of the original MLST protocol (16). Our workflow used primers for the MLST scheme, which amplifies the extended regions for each locus covering the positions of the original primer sequences and has an advantage in distinguishing between ST33 and ST14.

In the current MLST scheme for *M. pneumoniae*, differences between some STs were derived only from a single SNP in one of the eight MLST markers. For example, compared with ST3, ST19, ST20, and ST30 had only a single SNP at the *pgm*, *gmk,* and *gyrA* loci, respectively. The same was true for ST33 and ST14 as described above. Therefore, caution should be exercised when interpreting MLST differences in the phylogenetic classification of *M. pneumoniae* strains. The differences in MLST types due to slight genetic variations may lead to an overestimation of the phylogenetic relationship between strains. A combination of multiple genotyping methods is important to better understand the phylogenetic relationships of *M. pneumoniae*. In the genotyping workflow developed in this study, we combined *p1* typing, MLST, and SNP typing for the type 1 lineage (MPN295, MPN607, and MPN678), and the 23S rRNA gene for macrolide resistance. We believe that this workflow provides a useful and universal platform for *M. pneumoniae* genotyping.

This study has several limitations. The phylogenetic trees (Fig. 2A and B) were constructed using whole-genome sequence data of *M. pneumoniae* strains available in public databases, many of which originated from East Asia. Consequently, the data are possibly regionally biased. For example, the smaller number of non-Asian strains may contribute to the clustering of Asian strains, whereas non-Asian strains tend to group into distinct clusters separate from those of the Asian strains. A phylogenetic tree that includes more non-Asian strains might differ slightly from the one generated in this study. Additional publicly available reports from non-Asian countries would improve the accuracy of interpretation of the genetic relationships of *M. pneumoniae* strains. Another limitation is that the strategy is unsuitable for use with clinical samples because nested PCR is generally required for direct amplification of such samples and multiplexing is typically not used for nested PCR. However, a nested PCR scheme could be developed if the amplification targets the *p1* operon only. This approach has potential, as complete sequencing of the *p1* operon requires an amplicon-based strategy.

In conclusion, we demonstrated that the genotyping workflow developed in this study, based on amplicon sequencing, is useful for epidemiological studies of *M. pneumoniae*. It was possible to obtain genotyping data that could be compared with previous reports for a number of strains using a relatively simple process with a short turnaround time. The typing results were based on sequencing of genotyping markers and were reliable. Even if whole-genome sequence data of *M. pneumoniae* are acquired in the future and more useful genotyping markers are identified, these markers could easily be incorporated into this workflow. The proposed genotyping workflow in this study provides a basic platform for developing a universal genotyping protocol for future phylogenetic studies of *M. pneumoniae*.

## Data Availability

The raw whole-genome sequencing and amplicon sequencing data are available in NCBI/ENA/DDBJ under BioProject numbers PRJDB18338 and PRJDB18337, respectively. The accession numbers for the complete and draft genome sequences are shown in Table S2.

## SUPPLEMENTAL MATERIAL

Supplemental figures spectrum.02719-24-s0001.pdf Fig. S1 to S3.

Supplemental tables spectrum.02719-24-s0002.xlsx Tables S1 to S4.

ASM does not own the copyrights to Supplemental Material that may be linked to, or accessed through, an article. The authors have granted ASM a non-exclusive, world-wide license to publish the Supplemental Material files. Please contact the corresponding author directly for reuse.
