# Supplementary material for: Multiplex amplicon sequencing for the comprehensive genotyping of Mycoplasma pneumoniae
Source: Microbiol Spectr. 2025 May 22;13(7):e02719-24. doi: 10.1128/spectrum.02719-24 (PMC12210885; doi:10.1128/spectrum.02719-24)
Supplement: Supplemental figures — Fig. S1 to S3. [file spectrum.02719-24-s0001.pdf]

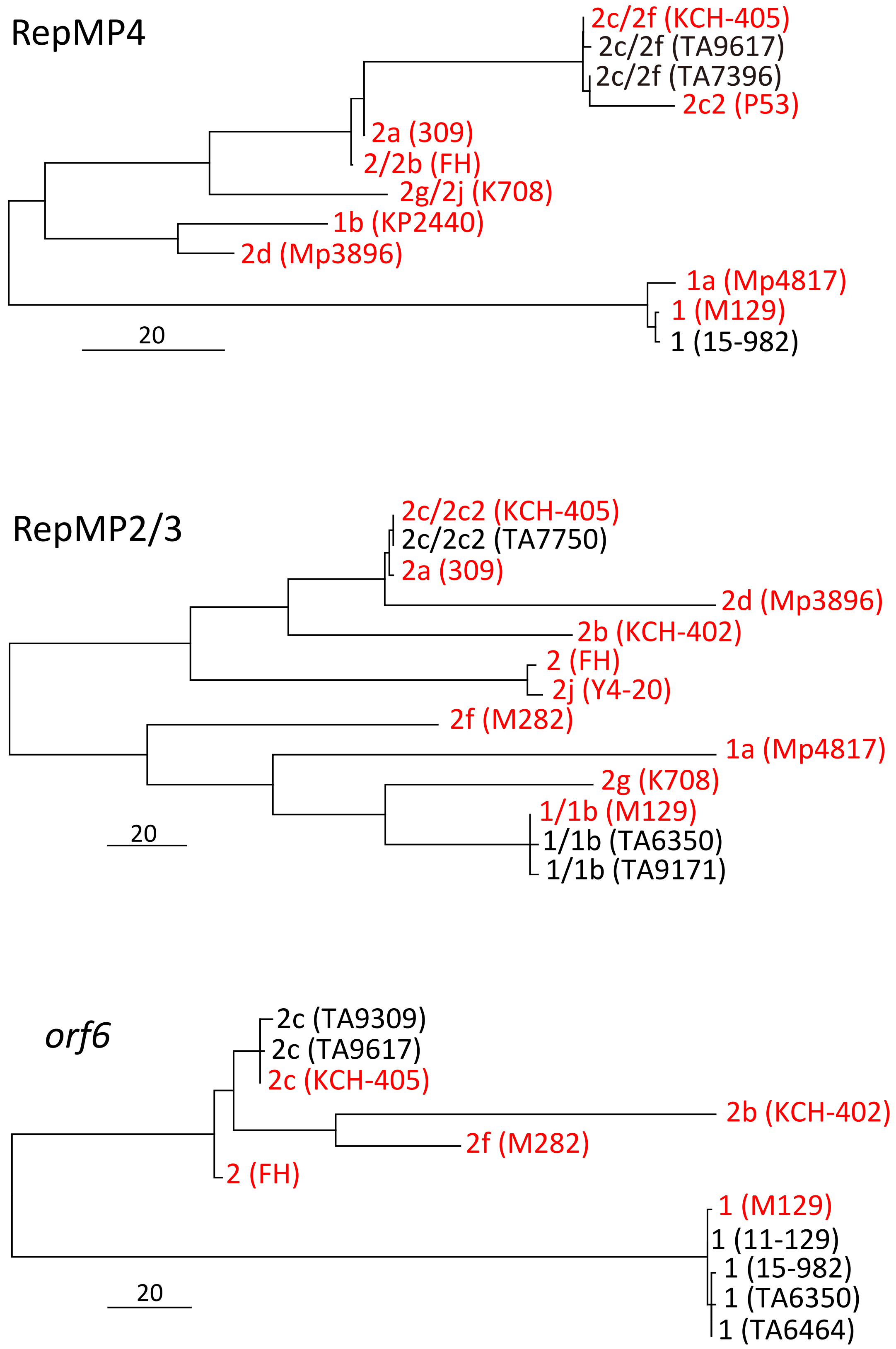

Figure S1. Non-rooted phylogenetic tree of the genotyping regions
A non-rooted neighbor-joining tree was created with the reference and detected sequences, written in red and black, respectively. The branch scale indicates the number of nucleotide differences.

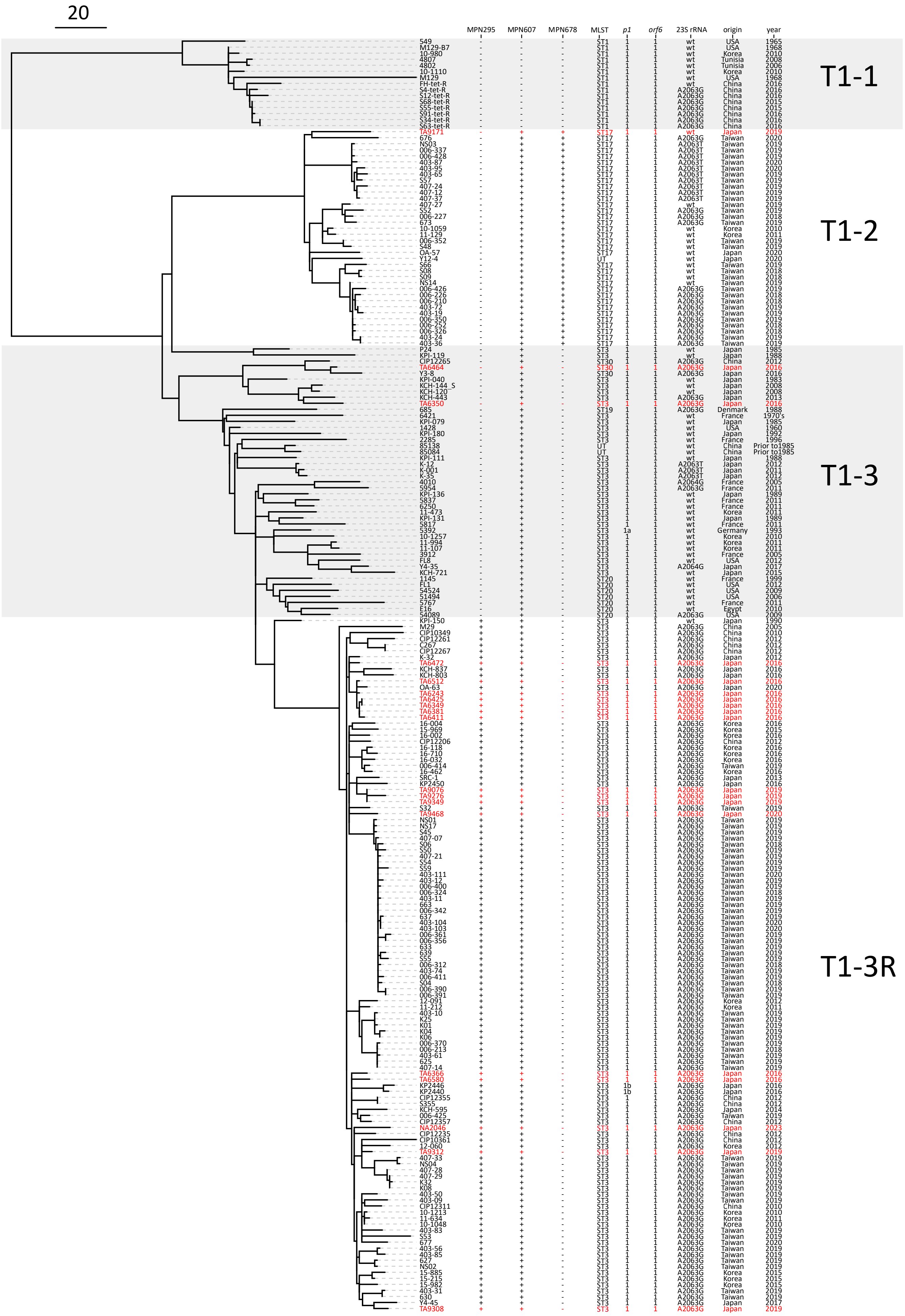

Figure S2. Whole-genome phylogenetic tree of *p1* type 1 lineage.
A non-rooted tree was created based on the SNPs of the 19 *p1* type 1 strains collected in Tokyo in this study, and 192 strains in the public database identified as *p1* type 1 lineage, with information for specifying each strain. The branch scale indicates the number of nucleotide substitution. SNP, single nucleotide polymorphism; UT, untypeable; wt, wild type.

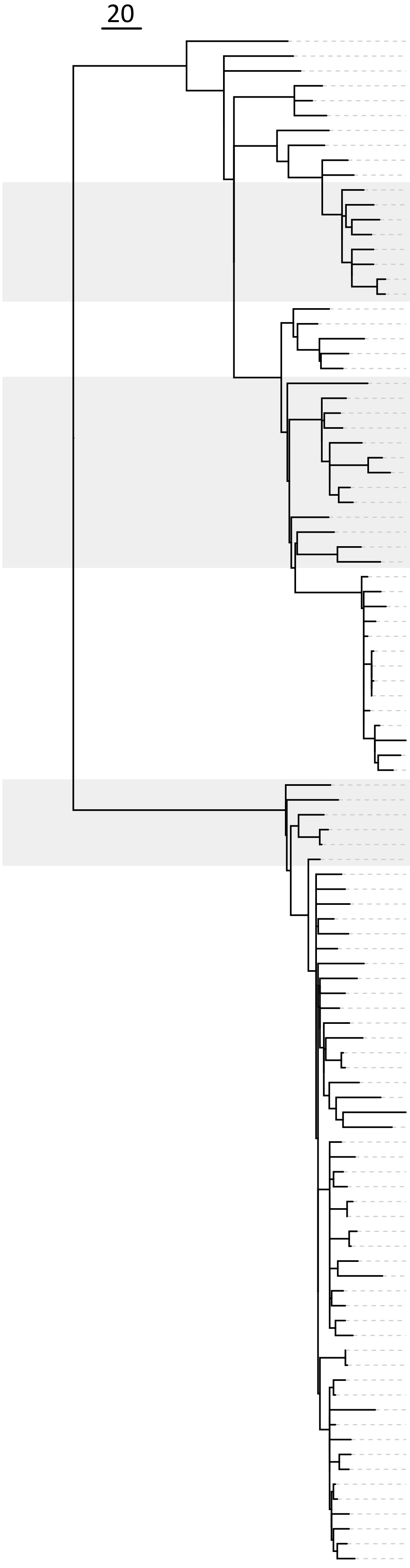

Strain	MLST	*p1*	*orf6*	23S rRNA	origin	year
MAC	ST2	2	2	wt	USA	1944
M1139	ST4	2	2	wt	England	1981
M547	ST2	2	2	wt	Denmark	1967
FH	ST2	2	2	wt	USA	1954
PO1	ST2	2	2	wt	USA	1980
2882	ST2	2	2	wt	Spain	1999
KPI-025	ST2	2	2	wt	Japan	1980
M2592	ST2	2	2	wt	England	1982
1134	ST2	2	2	wt	USA	1999
39443	ST2	2	2	wt	USA	1999
1801	ST2	2b	2b	wt	USA	2000
Y12-24	ST2	2b2	2b	A2063G	Japan	2020
KCH-402	UT	2b	2b	wt	Japan	2012
RI3	ST2	2b	2b	wt	USA	2007
CO103	ST2	2b	2b	wt	USA	2013
GA3	ST2	2b	2b	wt	USA	2012
6282	ST2	2b	2b	wt	France	2011
4911	ST2	2b	2b	wt	France	2008
M2192	ST47	2	2	wt	England	1982
KPI-072	ST47	2	2	wt	Japan	1985
3163	ST47	2	2	wt	France	2001
19294	ST47	2	2	wt	USA	1994
1006	ST47	2	2	A2063G	USA	1999
KPI-187	ST7	2	2	wt	Japan	1992
KPI-102	ST7	2	2	wt	Japan	1987
KPI-064	ST7	2	2	wt	Japan	1984
KPI-015	ST7	2	2	wt	Japan	1980
KPI-200	ST7	2	2	wt	Japan	1994
4358	UT	2	2	A2063C	Japan	2000-2003
4318	UT	2	2	C2617G	Japan	2000-2003
KPI-037	ST7	2	2	wt	Japan	1983
KPI-020	ST7	2	2	wt	Japan	1980
KPI-002	ST7	2	2	wt	Japan	1976
KPI-007	ST7	2	2	wt	Japan	1979
K27	ST16	2	2	wt	Kenya	2010
I-42	ST16	2	2	wt	Japan	2013
Y12-38	ST7	2j	2	wt	Japan	2020
K708	ST7	2g	2	wt	Japan	2016
TA9509	ST7	2j	2	wt	Japan	2020
TA9337	ST7	2j	2	wt	Japan	2019
TA9565	ST7	2j	2	wt	Japan	2020
TA9338	ST7	2j	2	wt	Japan	2019
TA9242	ST7	2j	2	wt	Japan	2019
TA9139	ST7	2j	2	wt	Japan	2019
TA9221	ST7	2j	2	wt	Japan	2019
TA8743	ST7	2j	2	wt	Japan	2019
Y4-67	ST7	2j	2	wt	Japan	2017
Y3-12	ST7	2j	2	wt	Japan	2016
Y4-20	ST7	2j	2	A2063G	Japan	2016
Y4-15	ST7	2j	2	wt	Japan	2016
5393	ST2	2a	2	wt	Germany	1991
KPI-199	ST2	2a	2	wt	Japan	1993
309	ST2	2a	2	wt	Japan	1998
KPI-165	ST2	2a	2	wt	Japan	1991
KPI-170	ST2	2a	2	wt	Japan	1991
519	ST15	2a	2	wt	USA	1995
KCH-338	ST14	2a	2c	wt	Japan	2011
CO3	ST14	2a	2c	wt	USA	2014
6009	ST14	2a	2c	wt	France	2011
3896	ST14	2d	2c	wt	France	2005
KCH-348	ST14	2a	2c	wt	Japan	2011
E57	UT	2a	2c	wt	Egypt	2009
KCH-266	ST14	2c	2c	wt	Japan	2010
TA6494	ST14	2c	2c	wt	Japan	2016
11-1384	ST33	2k	2c	wt	Korea	2011
11-949	ST14	2k	2c	A2063G	Korea	2011
KCH-161	ST14	2c	2c	wt	Japan	2009
TA9313	ST14	2c	2c	wt	Japan	2019
TA8574	ST14	2c	2c	wt	Japan	2018
TA8674	ST14	2c	2c	wt	Japan	2018
M282	ST14	2f	2f	wt	Japan	2016
KCH-501	ST14	2c	2c	wt	Japan	2013
KCH-825	UT	2c	2c	wt	Japan	2016
Y3-2	ST14	2a	2c	wt	Japan	2016
TA7750	ST14	2c	2c	wt	Japan	2018
TA9309	ST14	2c	2c	wt	Japan	2019
TA9307	ST14	2c	2c	wt	Japan	2019
006-380	ST14	2c	2c	wt	Taiwan	2019
403-88	ST14	2c	2c	A2063G	Taiwan	2020
403-06	ST14	2a	2c	A2063G	Taiwan	2019
006-268	ST14	2c	2c	wt	Taiwan	2018
S62	ST14	2a	2c	wt	Taiwan	2019
Y3-60	ST14	2c	2c	wt	Japan	2016
KCH-731	ST14	2c	2c	A2063G	Japan	2015
TA6463	ST14	2c	2c	wt	Japan	2016
TA9336	ST14	2c	2c	wt	Japan	2019
16-734	ST14	2c	2c	wt	Korea	2016
403-26	ST14	2c	2c	wt	Taiwan	2019
K-004_S	ST14	2c	2c	wt	Japan	2011
K-004_L	ST14	2c	2c	wt	Japan	2011
I-37	ST14	2c	2c	wt	Japan	2013
KCH-349	ST14	2c	2c	wt	Japan	2011
Y3-43	ST14	2c	2c	A2063G	Japan	2016
14-637	ST14	2c	2c	wt	Korea	2014
TA6778	ST14	2c	2c	wt	Japan	2017
TA9241	ST14	2c	2c	wt	Japan	2019
TA7396	ST14	2c	2c	wt	Japan	2017
11-174	ST14	2c	2c	wt	Korea	2011
10-1385	ST14	2c	2c	wt	Korea	2010
K-24	ST14	2c	2c	wt	Japan	2012
KCH-405	ST14	2c	2c	wt	Japan	2012
S13	ST14	2a	2c	wt	Taiwan	2018
TA9617	ST14	2c	2c	wt	Japan	2021

Figure S3. Whole-genome phylogenetic tree of *p1* type 2 lineage.
A non-rooted tree was created based on the SNPs of the 21 *p1* type 2 strains collected in Tokyo in this study, and 82 strains in the public database identified as *p1* type 2 lineage, with information for specifying each strain. The branch scale indicates the number of nucleotide substitution. The clades shown in shadowed windows are the same as those shown in Fig. 2. SNP, single nucleotide polymorphism; UT, untypeable; wt, wild type.
